# Recombinant GDF15 prophylaxis attenuates cardiorenal toxicity and fibrosis induced by doxorubicin and angiotensin II

**DOI:** 10.1186/s13578-026-01633-9

**Published:** 2026-08-16

**Authors:** Erik Fung, Hangqi Luo, Jack C. H. Chen, Wenjuan Zhu, Lihua Huang, Yidan Hao, Xiao-Ru Huang, Hui-Yao Lan, Ellen N. Poon

**Affiliations:** 1 School of Medicine, The Hong Kong University of Science and Technology, 5th floor, Academic Building, Clear Water Bay, Kowloon, Hong Kong SAR, China; 2 Laboratory for Heart Failure + Circulation Research, Li Ka Shing Institute of Health Sciences, and Gerald Choa Cardiac Research Centre, Faculty of Medicine, The Chinese University of Hong Kong, Hong Kong SAR, China; 3 Centre for Cardiovascular Genomics and Medicine and Hong Kong Hub of Paediatric Excellence, The Chinese University of Hong Kong, Hong Kong Children’s Hospital, Hong Kong SAR, China; 4 School of Public Health, Faculty of Medicine, Imperial College London, London, UK; 5 Yale Cardiovascular Research Center, Section of Cardiovascular Medicine, Yale School of Medicine, New Haven, CT USA; 6 School of Biomedical Sciences, Faculty of Medicine, The Chinese University of Hong Kong, Lo Kwee-Seong Integrated Biomedical Sciences Building, Area 39, Shatin, New Territories, Hong Kong SAR, China; 7 Stanford Cardiovascular Institute, Stanford University, Stanford, CA USA; 8 Inflammatory Disease Laboratory, Li Ka Shing Institute of Health Sciences, Faculty of Medicine, The Chinese University of Hong Kong, Prince of Wales Hospital, Hong Kong SAR, China; 9 Hong Kong Hub of Paediatric Excellence (HK HOPE), The Chinese University of Hong Kong, Hong Kong SAR, China; 10 Ministry of Education Key Laboratory for Regenerative Medicine, The Chinese University of Hong Kong, Hong Kong SAR, China

**Keywords:** Anthracycline, Cardiorenal toxicity, Angiotensin II, Growth differentiation factor 15

## Abstract

**Background:**

Anthracycline chemotherapy induces cardiotoxicity through mitochondrial damage and cell apoptosis that are exacerbated in vivo by angiotensin II (Ang II). Growth differentiation factor 15 (GDF15) is a stress-responsive cytokine with protective functions but its capacity to mitigate doxorubicin (Dox)-induced cardiotoxicity and Ang II-associated cardiorenal stress remains unclear.

**Methods:**

Human embryonic stem cell-derived cardiomyocytes were used to evaluate Dox toxicity and to benchmark recombinant GDF15 against conventional dexrazoxane pretreatment. Cardiomyocyte viability, mitochondrial integrity and function, apoptosis, and transcriptome profiles by RNA sequencing were evaluated. To determine in vivo efficacy, C57BL/6 J mice were administered GDF15 (0.5 mg/kg, intraperitoneal) 24 h before once weekly Dox (5 mg/kg). Ang II was infused subcutaneously via surgically implanted osmotic pump. Mice were sacrificed for histopathologic analysis 1 week after the fourth cycle of Dox.

**Results:**

In vitro, Dox-induced mitochondrial fragmentation, loss of membrane potential, transcriptional dysregulation, and apoptosis were significantly attenuated by recombinant GDF15 pretreatment, with efficacy comparable to dexrazoxane. Cytotoxic effects of daunorubicin on cardiomyocytes were also attenuated by GDF15. In vivo, Ang II, Dox, and Dox + Ang II induced cardiorenal injury characterized by hypertension, sarcomere disarray, myocardial and renal fibrosis, and glomerular, tubular, and perivascular abnormalities. GDF15 prophylaxis mitigated these pathologic changes, preserved myocardial and renal tissue architecture, and reduced cardiorenal fibrosis.

**Conclusions:**

Recombinant GDF15 pretreatment can protect the heart and kidneys from Dox-induced cardiorenal toxicity and Ang II-exacerbated injury. These findings encourage further investigation into GDF15 as a potential prophylaxis against cardiorenal toxicity associated with anthracycline chemotherapy and renin-angiotensin system activation.

**Supplementary Information:**

The online version contains supplementary material available at https://doi.org/10.1186/s13578-026-01633-9.

## Introduction

Anthracyclines (e.g. doxorubicin, daunorubicin) are chemotherapeutic agents that are frequently used in the treatment of soft tissue neoplasia and hematologic malignancies [1–3]. However, benefits of their anticancer action are offset by dose-dependent cardiotoxicity, resulting in cardiac dysfunction, cardiomyopathy, and heart failure in up to half of the exposed patients [1–7]. Although the risk of anthracycline-induced cardiotoxicity may be reduced by prophylactic dexrazoxane [1–3, 7], the topoisomerase-inhibiting iron chelator may increase the future risk of a secondary hematologic malignancy. [1, 8]

Hypertension and over-activation of the renin-angiotensin system are recognized risk factors for anthracycline-induced cardiotoxicity and cardiomyopathy [7, 9–11]. While angiotensin receptor blockade can reduce the risk of anthracycline-induced cardiotoxicity, its protective effect is modest [7, 9–11]. Biologic modulators are increasingly used for cardioprotection and growth differentiation factor 15 (GDF15) [12], an endogenous circulating protein with neurometabolic and immune modulating action, has been shown to counter adverse cardiac remodeling, hypertensive cardiomyopathy, and myocardial infarction [13–17]. However, its preclinical efficacy in vivo and its effects on the cellular and subcellular levels are largely uncharacterized in anthracycline-induced cardiotoxicity.

GDF15 is a transforming growth factor-β superfamily member encoded by the *GDF15* gene on chromosome 19p13.11 that is post-translationally processed into a 308-amino acid protein and released as a 25-kDa dimer in response to tissue injury, cellular stress, and inflammation [9–14]. While intensely studied as a biomarker of decompensated heart failure, GDF15 was found to remain elevated despite resolution of hemodynamic congestion and dissociated from trajectories of B-type natriuretic peptide (BNP) or N-terminal prohormone of BNP that directly indicated ventricular stress or stretch [18]. Meanwhile, experimental rodent models revealed that genetic insufficiency of *Gdf15* conferred increased myocardial hypertrophy and fibrosis, mitochondrial damage, oxidative stress, vascular dysfunction and apoptosis [13, 14], whereas tissue-specific overexpression of GDF15 protected against ventricular hypertrophy induced by phenylephrine and angiotensin II, and in a mouse model of pressure overload [13]. Angiotensin II is a key member of the renin–angiotensin–aldosterone axis that mediates cardiac and renal neurohormonal interactions in the pathophysiology of heart failure [19], and exacerbates anthracycline-induced cardiotoxicity [9–11]. Whether recombinant GDF15 can be harnessed to protect the heart and kidneys from anthracycline-induced toxicity remains untested. In this study, we determined in human embryonic stem cell-derived cardiomyocytes (hESC-CMs) the optimal condition for GDF15 pretreatment against the adverse effects of anthracycline-induced toxicity on mitochondrial function, cell survival and gene expression in vitro; benchmarked the efficacy of GDF15 against dexrazoxane; and evaluated in a mouse model the cardiorenal protective efficacy of prophylactic GDF15 against doxorubicin with or without the additional stress of angiotensin II.

## Methods

### Human embryonic stem cell culture and cardiac differentiation

**Fig. 1:**
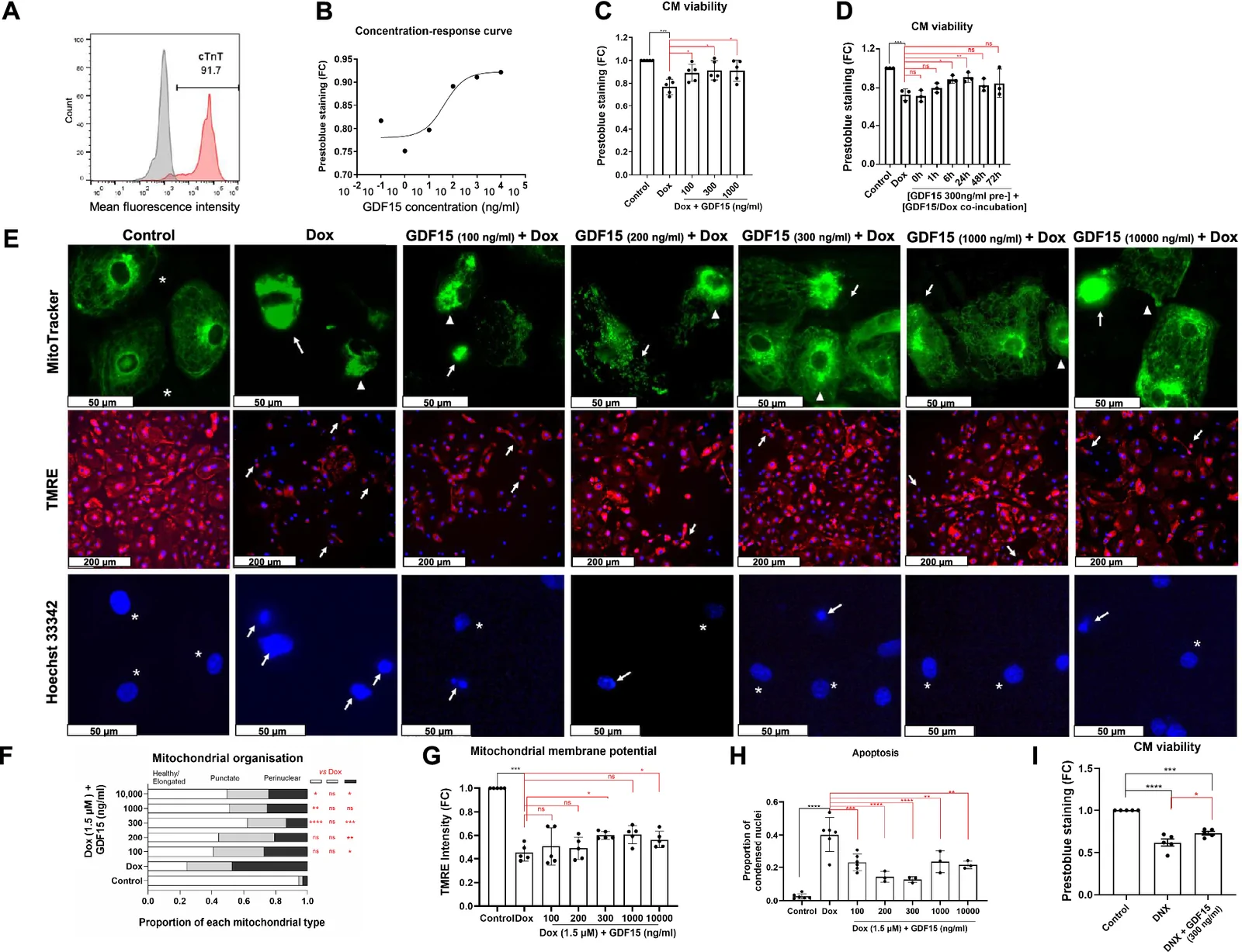
GDF15 prophylaxis protected hESC-CMs against doxorubicin (Dox). **A** Flow cytometry analysis of cardiac troponin T (cTnT)-positive cultured cells (red) relative to the isotype-matched negative control (grey) (n = 3). **B** Dose optimisation: hESC-CM viability was assessed by PrestoBlue after GDF15 pretreatment and subsequent incubation with Dox (1.5 µM) (n = 5). Data were normalised to control (equivalent volume of sterile H_2_O) and presented as fold change (FC). **C** Viability of hESC-CMs with GDF15 pretreatment at different concentrations (n = 5). **D** Pretreatment of hESC-CMs with recombinant GDF15 for 24 h improved cell viability upon addition of Dox (n = 3). **E** Fluorescence microscopy of mitochondrial network (first row, green, MitoTracker) revealed normal elongated structures (asterisk) and abnormal perinuclear (triangle) and punctate morphology (arrow) under different experimental conditions. Mitochondrial membrane potential (second row, red, TMRE) with depolarization (arrow) indicated functional impairment on merged imaging (blue nuclei, Hoechst 33,342). Normal (asterisk) and abnormal condensed nuclei (arrow) (third row, blue, Hoechst 33,342). Representative images from 3 to 6 independent experiments are shown. Mitotracker, TMRE and Hoechst images were captured at 100 × , 20 × and 100 × magnification respectively. Analysis of **F** mitochondrial organization (n = 3–6), **G** TMRE (n = 5) and **H** nuclei staining experiments (n = 3–6) are summarized. **I** hESC-CM viability was assessed by PrestoBlue assay after GDF15 pretreatment and subsequent incubation with daunorubicin (DNX, 2 µM) (n = 5). Sterile H_2_O was used as Control for the above experiments. Statistical significance was calculated using one-way ANOVA with Tukey post test (for Fig. 1C, D, E, F, I, J, and K), or two-way ANOVA with Tukey post hoc test (for Fig. 1H), and shown relative to control (Fig. 1E) and Dox-treated cells (Fig. 1C, D, F, H, I, J, and K). n, number of biological replicates. ns, non-significant. * p < 0^1^.05, ** p < 0.01, *** p < 0.001, **** p < 0.0001

H7 hESCs (Wicell) were cultured in E8 medium (Thermo Fisher Scientific) on hESC-qualified Matrigel-coated cell culture dishes (Corning) at 37 °C in a humidified 5% CO_2_ atmosphere. When cells reached ~ 80% confluency, denoted as day 0 (D0), cardiac differentiation [20] was induced with 6 μM CHIR99021 (Selleck Chemicals) in RPMI 1640 media supplemented with B-27 Supplement Minus Insulin (Thermo Fisher Scientific). On D2, the medium was changed to RPMI/B27 Supplement Minus Insulin, without CHIR99021. From D3 to D5, cells were cultured in RPMI/B-27 Supplement Minus Insulin containing 5 μM IWR-1 (Sigma-Aldrich), an inhibitor of the Wnt/β-catenin signaling pathway. Thereafter, RPMI/B-27 Supplement Minus Insulin was used until D7 when RPMI/B-27 (containing insulin) was used and refreshed every 2–3 days. By D7–D10, beating hESC-CMs were seen under light microscopy, with over 90% expressing cardiac troponin T on flow cytometric analysis (Fig. 1A) [21].

### Culture of MV4-11 cell line

MV4-11 leukemia cells were cultured in RPMI 1640 medium supplemented with 10% fetal bovine serum (FBS), 100 U/mL penicillin and 0.1 ng/mL streptomycin (Thermo Fisher Scientific), and incubated at 37 °C in a humidified atmosphere containing 5% CO_2_.

### Reagents and drugs

Recombinant human GDF15/MIC-1 protein (PeproTech), doxorubicin hydrochloride (Cayman Chemical), and dexrazoxane hydrochloride (Abcam, UK) were prepared according to manufacturers’ instructions. For animal experiments, lyophilized drugs were reconstituted in 0.9% saline to a final volume of 100 μL, and administered according to the required concentrations: doxorubicin, 5 mg/kg; GDF15, 0.5 mg/kg; dexrazoxane, 100 mg/kg. Angiotensin II (Sigma-Aldrich) was reconstituted in 0.9% saline and administered via subcutaneously implanted osmotic pump (see below). Daunorubicin (MedChemExpress, USA) was reconstituted in sterile water, and stock aliquots were kept in the dark at − 20 °C until addition to cell culture at a final concentration of 2 µM. For all in vitro experiments, the Control group used sterile H_2_O, whereas for all mouse experiments, the Control animals were injected with 0.9% normal saline.

### Dose response and time course experiments

To determine the optimal dose of GDF15 in enhancing the viability of hESC-CMs, 0.1, 1, 10, 100, 1,000 and 10,000 ng/ml of the recombinant protein was applied as pretreatment for 24 h before addition of 1.5 µM doxorubicin. Cell viability was assessed using the PrestoBlue assay (see below).

### Cell viability analysis by PrestoBlue assay

Cell viability was assessed using the PrestoBlue^TM^ assay (Thermo Fisher Scientific). Cells were seeded at a density of 15,000 cells per well on a 96-well flat-bottom microtiter plate and assayed when confluent. PrestoBlue was diluted 1:10 in culture medium and incubated with the cells for 30 min at 37 °C. Fluorescence intensity of PrestroBlue was measured using a Synergy HTX Multi-Mode microplate reader (Agilent Technologies) with 530 nm excitation and 590 nm emission.

### Analysis of mitochondrial morphology and nuclear condensation

hESC-CMs were incubated in RPMI/B27 supplement medium containing 50 nmol/L MitoTracker Deep Red (MTDR) (Thermo Fisher Scientific). After incubating with 1 µg/ml Hoechst 33,342 (Thermo Fisher Scientific) for 20 min, cells were visualized using a DMi8s Research Inverted Thunder Imager Live Cell System (Leica Microsystems, Germany) with excitation wavelength of 540 nm for MTDR and 480 nm for Hoechst 33,342. Mitochondrial morphology was classified into three types: elongated, punctate, and perinuclear, as described previously [22]. Nuclear morphology was categorized as condensed or normal.

### Detection of mitochondrial membrane potential

The cell-permeant cationic dye, tetramethylrhodamine ethyl ester (TMRE) (Thermo Fisher Scientific), was used to evaluate mitochondrial membrane potential. TMRE at 50 nM was added to RPMI/B27 media for 20 min, followed by detection of fluorescence using a Synergy HTX Multi-Mode microplate reader (Agilent Technologies) with excitation and emission wavelengths of 485 nm and 528 nm, respectively.

### Immunofluorescence staining

hESC-CMs were fixed in 4% paraformaldehyde for 15 min, and permeabilized using 0.1% Triton X-100 (Thermo Fisher Scientific) for 15 min. Following three washes in 1 × phosphate buffered saline (PBS), the cells were blocked with 10% FBS (Hyclone; Cytiva, USA) diluted in PBS and incubated for 1 h at room temperature. Cells were incubated overnight at 4 °C with primary antibody prediluted in 10% FBS in PBS, then washed three times with 1 × PBS, and incubated with a goat anti-mouse IgG1 secondary antibody (1:1,00 diluted in 10% FBS/PBS) (Thermo Fisher Scientific) for 1 h at room temperature. Finally, cells were washed twice with 1 × PBS and mounted using ibidi mounting medium with DAPI (Ibidi). Images were captured using a DMi8s Research Inverted Thunder Imager Live Cell System (Leica Microsystems, Germany) at 20 × , 40 × or 100 × magnification.

### RNA extraction, cDNA synthesis, and real-time quantitative PCR

Total RNA was extracted using TRIzol^TM^ reagent (Thermo Fisher Scientific). RNA quality was routinely assessed by spectrophotometry, with A260/280 ratios of approximately 2.0 for RNA and 1.8 for DNA. cDNA was synthesized from 0.5 μg of total RNA using PrimeScript^TM^ RT Master Mix (Takara, Japan). Briefly, the reverse transcription reaction was prepared on ice in a final volume of 10 μL containing 2 μL of 5 × PrimeScript^TM^ RT Master Mix and 0.5 μg of total RNA, with RNase-free dH_2_O added to volume. The reaction was performed at 37 °C for 15 min, followed by 85 °C for 5 s. Real-time quantitative PCR (qPCR) was performed using GoTaq® qPCR Master Mix (Promega, USA) on an Applied Biosystems QuantStudio 12 K Flex Real-Time PCR System (Thermo Fisher Scientific, USA). Each 10 μL reaction contained 5 μL of 2 × master mix, 0.3 μL primer mix, and 30 ng cDNA template. The amplification program consisted of an initial denaturation at 95 °C for 2 min, followed by 40 cycles of 95 °C for 3 s and 60 °C for 30 s. Amplification specificity was confirmed by melting curve analysis, which showed a single peak for each primer pair. Relative gene expression levels were calculated using the 2^−ΔΔCt^ method to determine fold changes between treatment and control groups. Primer sequences and amplicon sizes are provided in Supplementary Table S1 in accordance with MIQE 2.0 guidelines. [23]

#### RNA sequencing

Total RNA was isolated using ReliaPrep^TM^ RNA Tissue Miniprep System (Promega, USA), and checked for integrity before RNA sequencing (RNA-Seq). RNA-Seq was performed by Novogene Bioinformatics Technology (Beijing, China). In brief, the library was constructed using 1 μg of total RNA. A NextSeq 500 System (Illumina, USA) was used for sequencing to generate 100 bp paired-end reads. Quality trimmed reads were mapped onto the human genome (mm10) by Tophat2, and gene expression in terms of fragments per kilobase of transcript per million mapped reads of genes overlapping with gene annotations in Ensemble release 96 calculated by Cufflinks. The R/Bioconductor package DESEQ2 was used for detecting differentially expressed transcripts at 8 h. Three comparative analyses were done: Group 1, control vs. doxorubicin (Dox) only; Group 2, GDF15 and Dox (GDF15 + Dox) vs. Dox only; Group 3, dexrazoxane (Dex) and Dox (Dex + Dox) vs. Dox only. Three biological replicates were used for each condition. Differentially expressed genes (DEGs) were defined as log2 fold change of greater than 1 or less than − 1, and *p* < 0.05. Gene set enrichment analysis was done based on Gene Ontology (GO) analysis with a false discovery rate (FDR) of q ≤ 0.05.

#### Statement on animal research

The study was approved by the university's Animal Experimentation Ethics Committee, and complied with the University Bylaws, the relevant legislation of the Government of the Hong Kong Special Administrative Region, and the Hong Kong Code of Practice for the Care and Use of Animals for Experimental Purposes. Experiments were conducted in accordance with the guidelines of the Laboratory Animal Services Centre (http://www.lasec.cuhk.edu.hk) in line with the NIH Guide for the Care and Use of Laboratory Animals.

#### Measurement of blood pressure, heart weight and tibia length in mice

Blood pressure was measured at the indicated times using CODA® High Throughput System (Torrington, Connecticut, USA), a noninvasive blood pressure measurement system. Briefly, O-cuffs and volume pressure recording cuffs were pretested, and at least three measurements were made when the animals appeared calm and comfortable. Upon completion, the animals were returned to their cages. At the end of the experiment, all mice were euthanized using sodium pentobarbital 100 mg/kg administered intraperitoneally. The hearts were excised and weighed immediately. The tibia of each mouse was carefully dissected and its length measured. Heart size was normalized to tibia length expressed as a ratio of heart weight to tibia length (HW/TL, mg/mm).

#### Mouse model of doxorubicin-induced cardiotoxicity

10- to 12-week-old C57BL/6 J male mice were purchased from the university's Laboratory Animal Services Centre. The animals had free access to water and standard diet, and were kept to a 12 h/12 h light/night cycle in a specific pathogen-free facility at the Laboratory Animal Services Centre Research Unit at Prince of Wales Hospital, Hong Kong. To test the effects of GDF15 or dexrazoxane, mice were pretreated with either prophylactic agent via intraperitoneal injection at a dosage and frequency previously shown to elicit the relevant biological or clinical response. Intraperitoneal injection of recombinant GDF15 at 0.5 mg/kg, a dosage shown to induce myokine effects [24], was administered 24 h before doxorubicin (5 mg/kg). Dexrazoxane at 100 mg/kg was given 1 h before doxorubicin (5 mg/kg) to achieve a doxorubicin-to-dexrazoxane ratio of 1:20, as per clinical use [25]. To induce anthracycline-induced cardiotoxicity in C57BL/6 J mice, 5 mg/kg of doxorubicin was injected intraperitoneally at 1-week interval for 4 weeks, emulating 4 cycles of anthracycline chemotherapy administered at 60 mg/m [2] to human patients [26, 27]. All injectable reagents were freshly reconstituted in sterile 0.9% saline. In both pretreatment arms, the animals were sacrificed for histopathologic examination one week after the fourth cycle of doxorubicin.

#### Implantation of osmotic pumps

Animal surgery was performed in a designated sterile surgical area under aseptic technique in a calm and quiet environment of a research laboratory. Before surgery, an anesthetic mix of ketamine (80 mg/kg) and xylazine (10 mg/kg) in sterile normal saline was administered to the subject animal via intraperitoneal injection. Under anesthesia, the target incision site on the back of the immobilized animal was shaved and disinfected with 70% ethanol. The surgical field was secured and a 1-cm incision was made into the skin on the back of the mouse. By blunt dissection using a hemostat, a small pocket was created for subcutaneous implantation of an ALZET (Campbell, California, USA) osmotic miniature pump (1 cm × 0.3 cm). Pre-sterilized osmotic pumps were used. For the control group, osmotic pumps contained normal saline only. For animals receiving angiotensin II, osmotic pumps were pre-filled with 1.4 mg/ml angiotensin II (Sigma-Aldrich) reconstituted in 0.2 ml of normal saline. Each animal received one osmotic pump inserted into the pocket, and the wound was closed with sterile wound clips. Animals implanted with angiotensin II- or saline-filled osmotic pumps were then subjected to doxorubicin with or without GDF15 or dexrazoxane prophylaxis. Postoperative buprenorphine (0.05 mg/kg) injection was immediately administered after wound closure and as needed before the animals recovered from anesthesia. After surgery, the animals were kept warm in a temperature-controlled holding apparatus to maintain stable body temperature until they regained consciousness and appeared fully awake, whereupon they were returned to the animal room with free access to water and food. Animals were monitored daily. Wound clips were removed 10 days after surgery.

#### Hematoxylin and Eosin staining of tissue sections

Murine hearts were collected after sacrifice and fixed in 4% paraformaldehyde overnight, followed by embedding in paraffin. Hearts were cut into 8-μm thick sections and immobilized on glass slides. In preparation for hematoxylin and eosin (H&E) staining, the sections were deparaffinized and sequentially rehydrated in 100%, 95% and 70% alcohol each for 15 min. After deparaffinization and rehydration, the slides were placed in Hematoxylin Solution (Mayer’s; Sigma-Aldrich), incubated for 5 min, then rinsed with dH_2_O followed by acid alcohol treatment for 10 s to remove excess staining dye, and repeated once again. Afterwards the slides were placed in Eosin Y Solution (Sigma-Aldrich) for 2 min, rinsed twice with dH_2_O, followed by dehydration in absolute alcohol. Finally, the slides were mounted using the Permount^TM^ medium (Thermo Fisher Scientific).

#### TUNEL staining of tissue sections

Terminal deoxynucleotidyl transferase (TdT) dUTP nick end labelling (TUNEL) staining for apoptosis was performed according to manufacturer's instructions (Abcam, UK). Sections were deparaffinized and rehydrated as above discussed. Afterwards sections were permeabilized with Proteinase K solution (1:100 in dH_2_O), quenched with 3% H_2_O_2_, incubated at 37 °C for 5 min, and equilibrated in 100 μL TdT Equilibration Buffer for 30 min at 37 °C. The labeling reaction was performed using 40 µL TdT-labelled reaction mix at 37ºC for 1.5 h. Reaction was stopped with 100 μL Stop Buffer at room temperature for 5 min. 100 μL of Blocking Buffer was then applied at room temperature for 10 min, followed by incubation with 100 μL 1 × conjugate at room temperature for 30 min. Each specimen was covered with 100 μL diluted DAB solution and incubated at room temperature for 15 min. Finally, the slides were rinsed with dH_2_O and mounted using Permount^TM^ medium.

#### Masson’s trichrome staining of tissue sections

Tissue sections were deparaffinized and rehydrated in preparation for staining. First, sections were carefully rinsed under running tap water for 10 min and washed once in dH_2_O. Sections were then stained in Biebrich Scarlet Acid Fuchsin Solution (Sigma-Aldrich) for 15 min, washed once in dH_2_O, then covered with phosphomolybdenum-phosphotungstic acid solution (Sigma-Aldrich), and incubated for 15 min at room temperature. Next, the solution was discarded and directly placed in aniline blue solution for 10 min. Sections were rinsed once with dH_2_O, incubated in 1% acetic acid solution for 5 min, and rinsed again in dH_2_O. Sections were then dehydrated in a series of quick passages through 95% ethyl alcohol, absolute ethyl alcohol, and xylene. Slides were mounted using Permount^TM^ medium.

#### Periodic acid-schiff staining of kidney tissue

The standard procedure of the Sigma-Aldrich periodic acid-Schiff staining system was used as per manufacturer's instructions. Briefly, kidney tissue sections were deparaffinized and hydrated to deionized water. After immersion of slides in Periodic Acid Solution for 5 min at room temperature, they were rinsed multiple times with distilled water and placed in Schiff's Reagent for 15 min at room temperature. Afterwards the slides were rinsed in running tap water for 5 min, counterstained in Hematoxylin Solution (Gill No. 3) for 90 s, and rinsed again in running tap water. The slides were allowed to dehydrate by air drying, and mounted with Permount Mounting Medium (Biolyst, USA).

#### Data and statistical analysis

ImageJ version 1.53 k and Pannoramic 250 FLASH III Digital Scanner with SlideViewer software version 2.9 (3DHISTECH, Hungary) were used for quantitative analysis of cytological and histological images. For statistical analysis, SPSS version 15.0 and GraphPad PRISM 10 software were used. Normality was evaluated using a Shapiro–Wilk normality test. For normally distributed data, results were analyzed using Student’s* t* test for paired comparisons, and one-way or two-way ANOVA followed by Tukey and Dunnett's post hoc tests for multiple-group comparisons, as appropriate. For non-Gaussian distribution, data were analyzed using nonparametric Friedman or Kruskal–Wallis test. The mean, standard error of the mean, and p-values were calculated. A* p*-value of < 0.05 was considered to be statistically significant.

## Results

### Pretreatment dosing of GDF15 as a protective agent against doxorubicin, and evaluation of mitochondrial network, membrane potential and apoptosis in hESC-CMs

hESC-CMs were differentiated via modulation of the Wnt signaling pathway and cultured for 60–120 days before experimentation. Over 90% of the cells expressed cardiac troponin T (Fig. 1A) and exhibited spontaneous beating. Following exposure to doxorubicin (1.5 µM, ~ C_max_ of this drug in patients), their viability fell by ~ 20%. Pretreatment of hESC-CMs with GDF15 at 100–1,000 ng/ml (optimally, 300 ng/ml) for 24 h before exposure to doxorubicin significantly improved cell viability (Fig. 1B–D).

Addition of 1.5 µM doxorubicin disrupted mitochondrial network (Fig. 1E and F), as shown by a decreased proportion of healthy elongated mitochondria and increased proportion of unhealthy punctate and perinuclear mitochondria. Doxorubicin also reduced the intensity of the potentiometric mitochondrial dye, TMRE, suggesting a reduction in mitochondrial membrane potential (Fig. 1E and G), and induced condensation and fragmentation of nuclei consistent with apoptosis (Fig. 1E and H). GDF15 pretreatment significantly improved mitochondrial network integrity (Fig. 1E and F) and membrane potential (Fig. 1E and G), and reduced nuclear condensation (Fig. 1E and H). GDF15 pretreatment also improved the viability of hESC-CMs against daunorubicin (*p* < 0.05, Fig. 1I), another commonly used anthracycline.

### Effects of GDF15 alone, or with doxorubicin, on the viability of MV4-11 leukemia cells

**Fig. 2:**
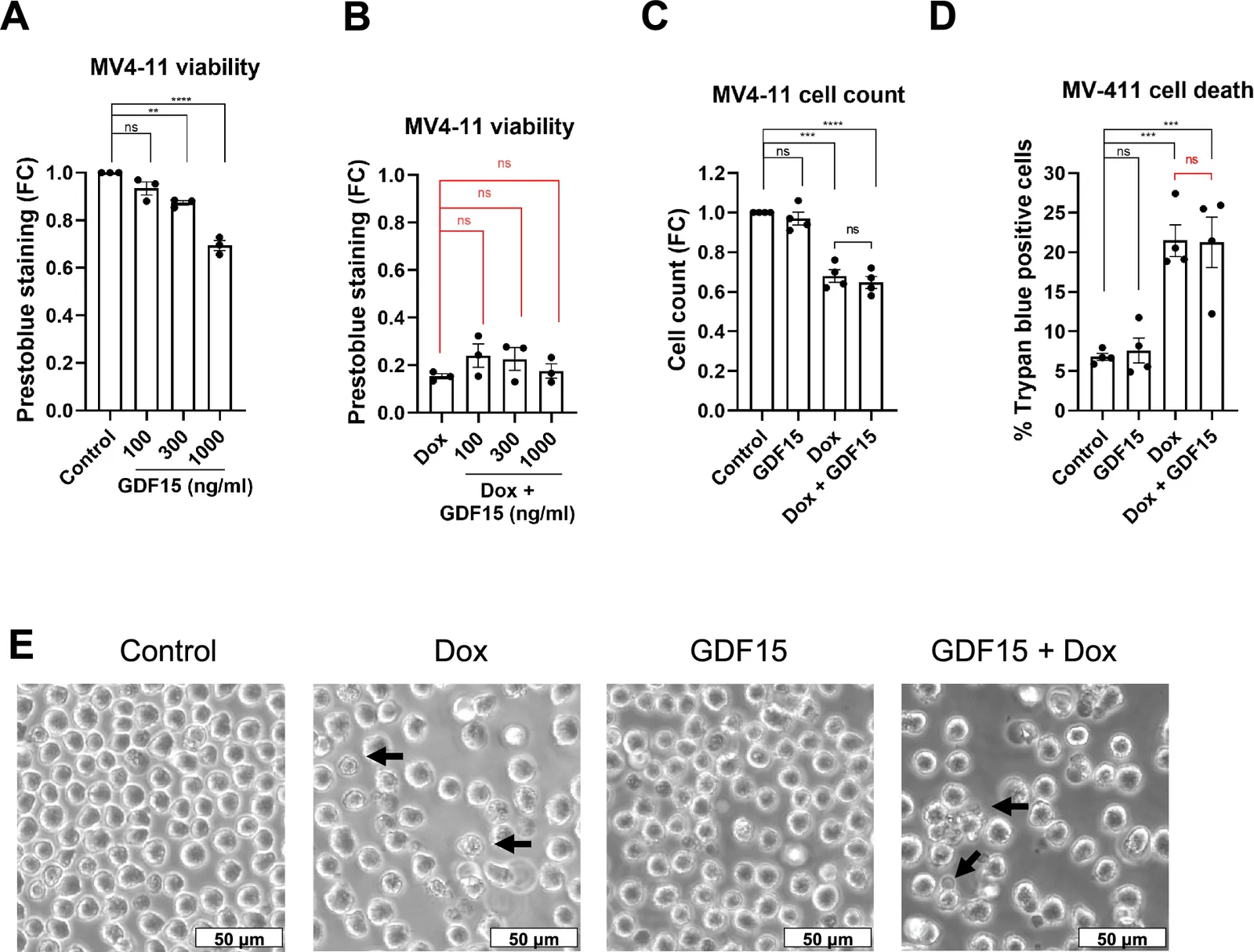
GDF15 alone, or with doxorubicin (Dox), did not alter MV4-11 leukemia cell number or the percentage of trypan blue-positive dead cells. Viability of MV4-11 leukemia cells were assessed at different concentrations of **A** GDF15 alone for 24 h, or **B** GDF15 pretreatment for 24 h followed by Dox (0.2 µM) (n = 3). **C** Total cell count was normalized to that of control cells, enumerated using hemocytometry, and presented as fold change (FC) and **D** the percentage of trypan blue-positive cells quantified (n = 4). **E** Representative fields of MV4-11 cells from the control and treatment groups are visualized under phase contrast microscopy (n = 4). Sterile H_2_O was used as Control for the above experiments. Arrows indicate dead or dying cells. n, number of independent experiments. ns, non-significant. ** p < 0.01, *** p < 0.001, **** p < 0.0001

To assess if GDF15 had oncogenic potential recognized as a problem associated with dexrazoxane [1, 8], we applied the recombinant protein only to cultured MV4-11 leukemia cells, or as a 24-h pretreatment before adding doxorubicin, and assessed for cell viability and cell count. GDF15 dose-dependently reduced the viability of MV4-11 leukemia cells (*p* < 0.01 and *p* < 0.0001 for 300 and 1,000 ng/ml of GDF15, respectively) (Fig. 2A), and did not protect cancer cells against doxorubicin (all *p* = ns for GDF15 at 100, 300 and 1,000 ng/ml) (Fig. 2B). Compared with the control, GDF15 only did not alter the total cell count. Doxorubicin alone effectively killed MV4-11 leukemia cells, and GDF15 pretreatment for 24 h did not alter the cell count or percentage of dead cells (Fig. 2C–E).

### Benchmarking GDF15 against dexrazoxane in hESC-CMs revealed comparable efficacy

**Fig. 3:**
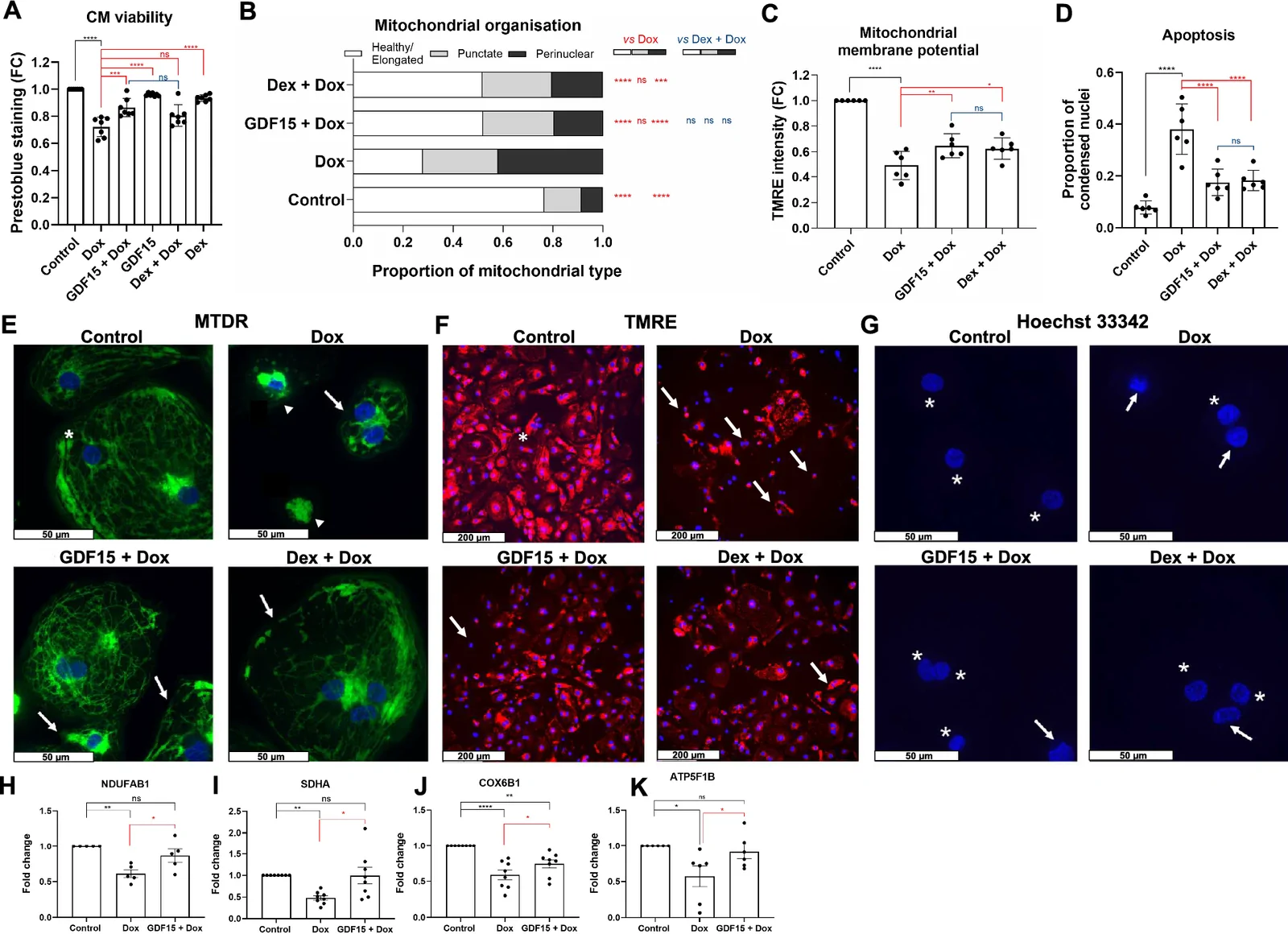
GDF15 and dexrazoxane (Dex) exerted similar protective effects on cultured hESC-CMs. Comparison of GDF15 with dexrazoxane (Dex) pretreatment before exposure to Dox (1.5 µM) showed similar **A** hESC-CM viability (n = 7); **B** and **E** mitochondrial network integrity and morphology (green; elongated, asterisk; perinuclear, arrow; punctate, triangle) (n = 6); **C** and **F** mitochondrial membrane potential (red; normal, asterisk; depolarized, arrow) (n = 6); **D** and **G** nuclear morphology and condensation indicative of apoptosis (blue; normal, asterisk; condensed nuclei, arrow) (n = 6). **H**–**K** qPCR analysis of mitochondrial genes (*NDUFAB1* (n = 5), *SHDA* (n = 5), *COX6B1* (n = 6) and *ATP5F1B* (n = 6)) normalized to GAPDH expression and control, and presented as fold change (FC). Sterile H_2_O was used as Control for the above experiments. Images were captured at 100 × , 20 × and 100 × magnification for Fig. 3E, 3F and 3G, respectively. Statistical significance was calculated using one-way (Fig. 3A, C, D, H) or two-way ANOVA (Fig. 3B) with Tukey post hoc test, and shown relative to Dox-treated cells and GDF15 + Dox vs. Dex + Dox (Fig. 3A–D). n, number of biological replicates. ns, non-significant. * p < 0.05, ** p < 0.01, *** p < 0.001, **** p < 0.0001

The effects of GDF15 was benchmarked against conventional dexrazoxane [11] pretreatment (Dex + Dox vs. Dox, *p* = ns) (Fig. 3A) at a clinically relevant dose of 200 µM for 1 h (mean peak plasma concentration of ~ 36.5 mg/L or 137 µM at 15 min following intravenous dexrazoxane infusion at 500 mg/m [2]) [25]. GDF15 pretreatment (GDF15 + Dox vs. Dox, *p* < 0.001) significantly improved cell viability (Fig. 3A). Mitochondrial organization (Fig. 3B and E) and membrane potential (GDF15 + Dox vs. Dox, *p* < 0.01; Dex + Dox vs. Dox, *p* < 0.05) (Fig. 3C and F), and apoptosis (both *p* < 0.0001, Fig. 3D and G) were improved by GDF15 or dexrazoxane to similar extents. Doxorubicin-induced suppression of mitochondrial genes, including complex I (*NDUFAB1*), II (*SDHA*), IV (*COX6B1*) and V (*ATP5F1B*) of the electron transport chain, were relieved by GDF15 (Fig. 3H–K).

### RNA sequencing analysis of gene expression alterations by GDF15 or dexrazoxane in the presence of doxorubicin

**Fig. 4:**
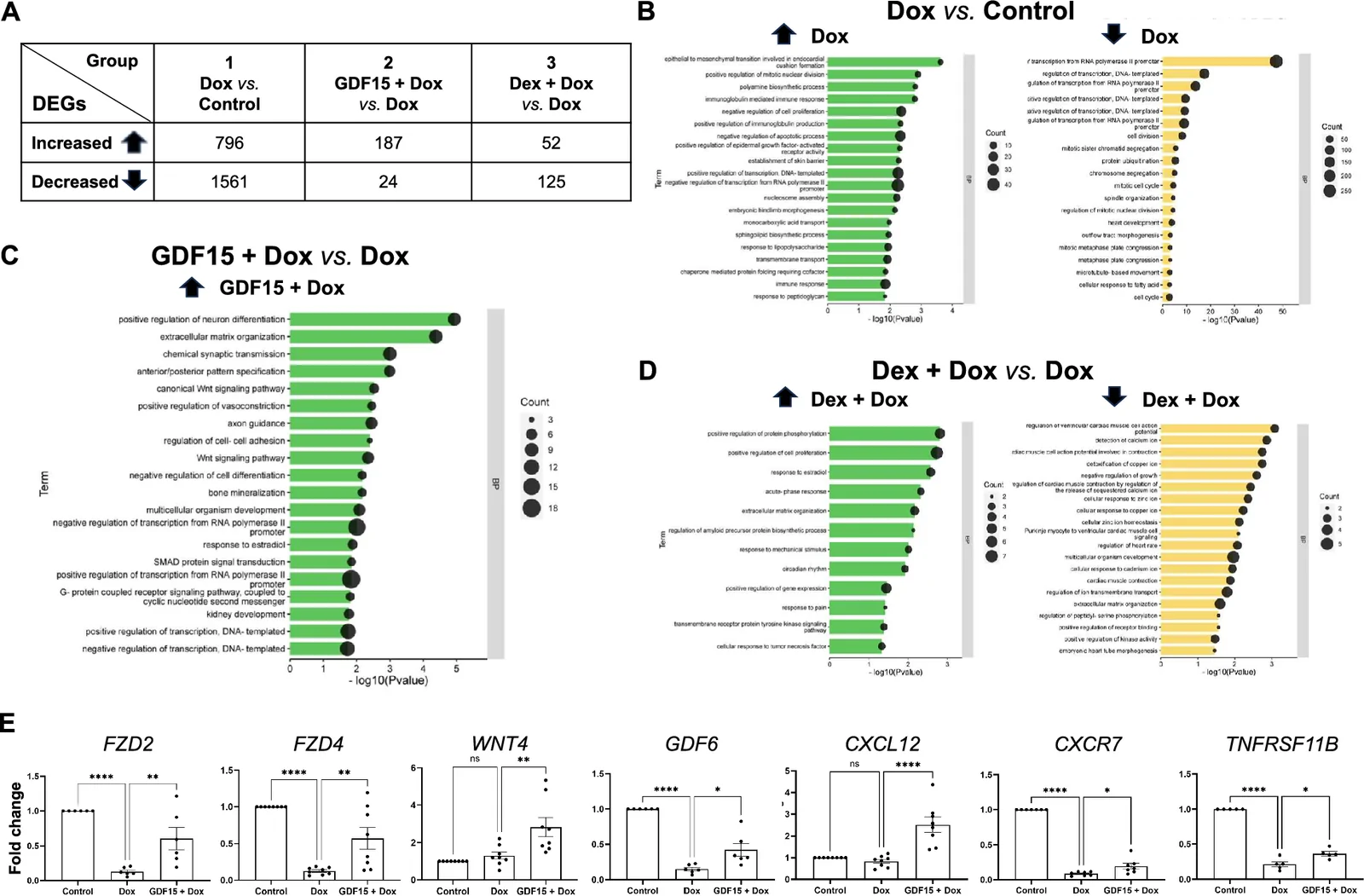
RNA sequencing results of hESC-CMs treated with doxorubicin, GDF15 and dexrazoxane. hESC-CMs were pretreated with GDF15 (300 ng/ml for 24 h) or dexrazoxane (Dex, 200 µM for 1 h), followed by doxorubicin treatment (Dox, 1.5 µM for 8 h). Sterile H_2_O was used as Control. **A** Summary of changes in expression of DEGs from DESEQ2 analysis based on a fold-change threshold of ≥ 2 and statistical significance level of *p* ≤ 0.05. **B**–**E** GO analysis was performed using a FDR threshold of q ≤ 0.05 to compare DEGs from three biological replicates in each group: (1) Dox vs. Control; (2) GDF15 + Dox versus Dox; and (3) Dex + Dox versus Dox. **F** qPCR validation of select DEGs (n = 6–8 independent experiments for each gene). Results were normalized to GAPDH expression and control, and presented as fold change (FC). Statistical significance was calculated using one-way ANOVA with Dunnett post-hoc test relative to Dox-treated cells. n, number of biological replicates. ns, non-significant. * p < 0.05, ** p < 0.01, **** p < 0.0001

To elucidate the gene expression patterns and molecular pathways modulated by GDF15 and dexrazoxane, hESC-CMs were pretreated with GDF15 or dexrazoxane as described, followed by incubation with doxorubicin for 8 h and analyzed by RNA sequencing. DEGs defined using DESEQ2 analysis and gene enrichment analysis was performed using gene ontology. Doxorubicin treatment significantly upregulated 796 genes and downregulated 1561 genes, relative to the control (Group 1, Dox vs. Control; Fig. 4A). Notably, genes involved in negative regulation of cell proliferation were upregulated, whereas those related to transcription were suppressed (Fig. 4B), consistent with doxorubicin’s known inhibition of topoisomerase activity [28]. Proapoptotic genes encoding known mediators of doxorubicin-induced cardiotoxicity including *TP53* [29] and *FAS* [30] were increased after doxorubicin treatment, while antiapoptotic genes including *MMP11*, *CXCL7*, and *PIK3R1* were decreased. Reduced expression of genes important for heart development (*HAND2*, *IRX4* and *NKX2-5)*, [31–33] and cellular response to fatty acid (*CREB1*) [34] and insulin (*IRS1*) [35] were also observed, suggesting compromised cardiac metabolism and repair.

Pretreatment with GDF15 prior to doxorubicin exposure when compared with Dox alone (Group 2, GDF15 + Dox vs. Dox) upregulated 187 and downregulated 24 genes (Fig. 4A). GO analysis revealed enhanced SMAD signal transduction (Figs. 4C), consistent with available evidence of GDF15 signaling via SMAD to regulate the TGF-β pathway [14]. Genes associated with other prosurvival pathways including Wnt signaling (*FZD4*, *GDF6*, *TCF7*, *FZD2*, *GDF7*, *WNT4*) were also increased. It is known that TGF-β and Wnt pathways interact in a network to regulate tissue homeostasis [36, 37]. Activation of Wnt via overexpression of *FZD2* and *FZD4* have been shown to promote cell survival and reduce apoptosis [38, 39]. GDF6, a component of SMAD signaling known to inhibit apoptosis [40], was upregulated by GDF15 pretreatment. GO analysis of downregulated DEGs did not result in significant pathway enrichment.

**Fig. 5:**
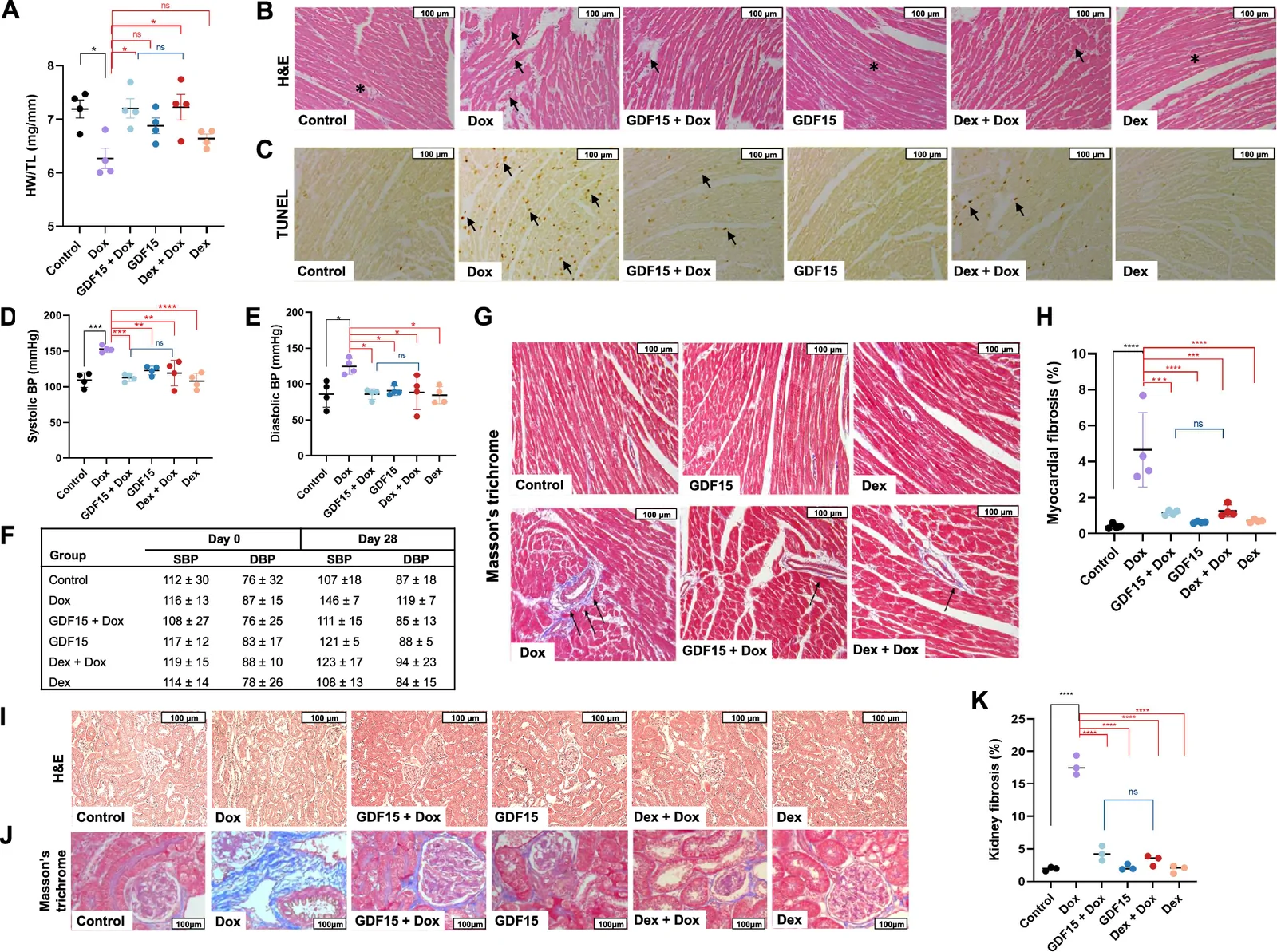
Mice prophylactically injected with GDF15 had attenuated doxorubicin (Dox)-induced cardiorenal toxicity. GDF15 or dexrazoxane (Dex) pretreatment was administered intraperitoneally before doxorubicin (Dox, 5 mg/kg) injection. Control animals were injected with 0.9% saline. Injections were given once a week for four weeks and mice were sacrificed 1 week after the fourth cycle of Dox. **A** Heart weight (HW) was indexed to tibia length (TL), and expressed as a ratio, HW/TL. **B** H&E staining of heart tissue revealed normal (asterisk) and abnormal (arrow) architecture. **C** TUNEL staining showed extensive cardiac cell apoptosis (arrow) in the Dox group, and reduced apoptosis in the GDF15 + Dox, and Dex + Dox pretreatment groups. **D**–**F** Systolic (S) and diastolic (D) blood pressure (BP) were measured. **G** and **H** Cardiac fibrosis (blue) was revealed by Masson’s trichrome staining and quantified using ImageJ software. **I** H&E staining of kidney tissue showed glomerular and tubular abnormalities, and **J** fibrosis (blue). Control, normal saline. Statistical significance was calculated using the one-way ANOVA with Tukey post hoc test, and shown relative to Dox-treated animals and GDF15 + Dox vs. Dex + Dox. n = 4 animals were used for each experiment. ns, non-significant. Scale bar = 100 µm. * p < 0.05, ** p < 0.01, *** p < 0.001, **** p < 0.0001

The protective effects of dexrazoxane were also evaluated in comparative analysis of Dex + Dox versus Dox. 52 and 125 genes were upregulated and downregulated, respectively (Group 3; Fig. 4A). Among the significantly upregulated genes associated with promotion of proliferation were *IL6R* and *NTRK2*, [41, 42] whereas the downregulated genes including *MT2A*, *MT1E* and *MT1F* [43–45] were associated with growth suppression and apoptosis (Fig. 4D); consistent with the known protective effects of dexrazoxane in our in vitro and in vivo experiments (Figs. 3 and 5). Genes involved in action potential regulation were downregulated. Expression changes of select candidate genes were confirmed by qPCR (Fig. 4E).

### GDF15 or dexrazoxane prophylaxis ameliorated doxorubicin-induced cardiorenal injury and fibrosis in mice

To determine the cardioprotective effects of GDF15 in vivo, we compared its prophylactic effects against those of dexrazoxane in a mouse model of anthracycline-induced cardiotoxicity. In response to doxorubicin only, the subject animals' heart sizes, expressed as an indexed HW/TL ratio, were significantly reduced (Fig. 5A). Doxorubicin induced myofibril loss and disarray (Fig. 5B), and increased cardiomyocyte apoptosis (Fig. 5C). The damaging effects of doxorubicin were improved by GDF15 or dexrazoxane pretreatment to similar extents (Fig. 5A–C). Doxorubicin-induced increases in systolic and diastolic blood pressure at D28 (Fig. 5D–F) and percentage of myocardial fibrosis (Fig. 5G and H) were also reduced by GDF15 (*p* < 0.001, *p* < 0.05 and *p* < 0.001, respectively) or dexrazoxane (*p* < 0.01, *p* < 0.05 and *p* < 0.001, respectively). Histopathologic analysis of the kidneys showed improvement in periglomerular and interstitial fibrosis in mice that received GDF15 or dexrazoxane pretreatment before doxorubicin (Fig. 5I–J).

### Angiotensin II-exacerbated hypertension and cardiorenal fibrosis from doxorubicin was attenuated by GDF15

**Fig. 6:**
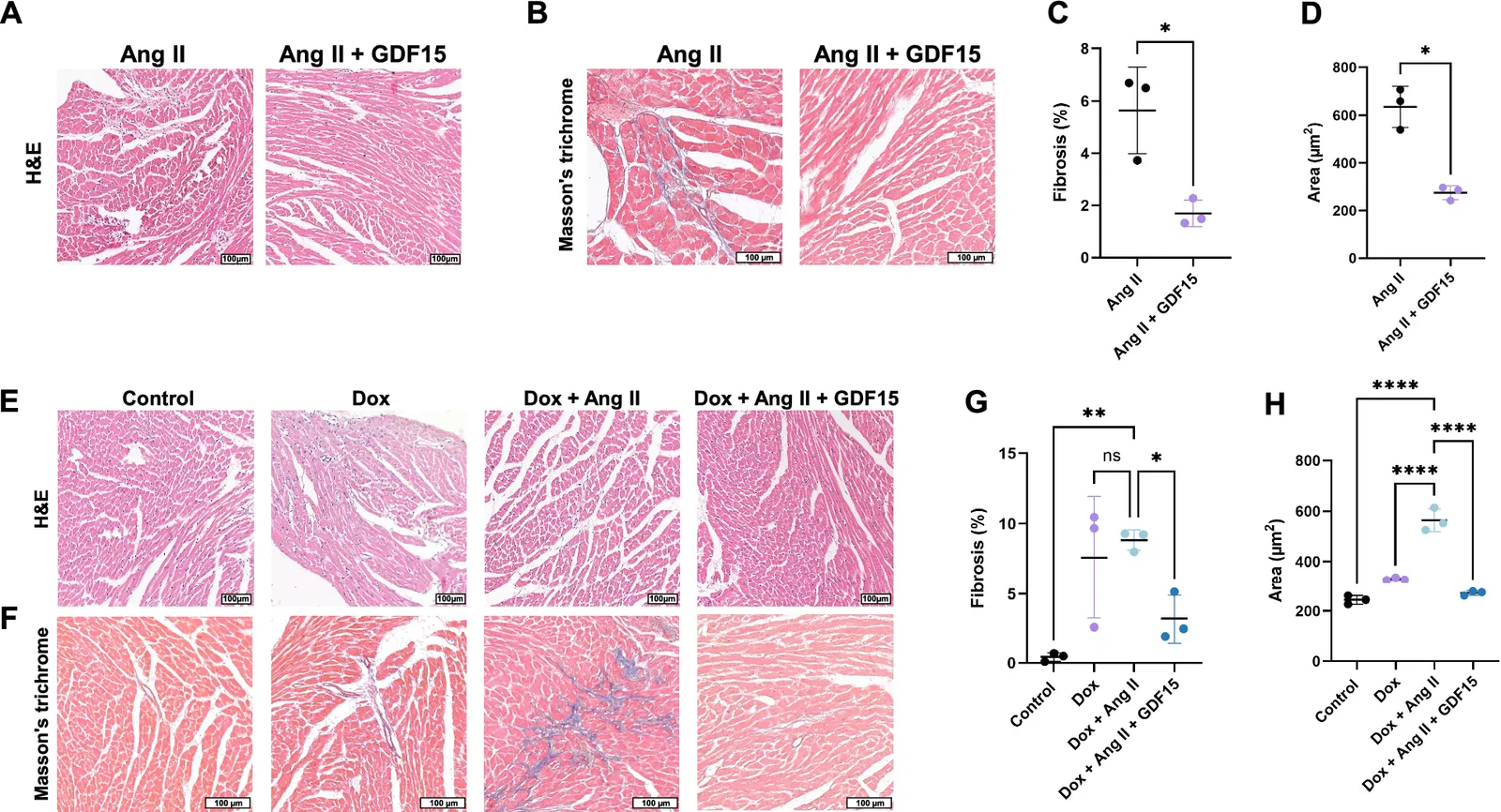
Cardiotoxicity induced by angiotensin II (Ang II) and doxorubicin (Dox) can be reduced by GDF15 pretreatment in mice. Mice were treated with Ang II ± GDF15. Control animals were injected with 0.9% saline. Heart tissue sections were stained with **A** H&E, and **B** and **C** Masson’s trichrome. **D** Cross-sectional cell area was quantified using imaging software. Mice were treated with vehicle control, Dox ± Ang II ± GDF15. Heart sections were subjected to **E** H&E, **F** and **G** Masson’s trichrome staining, and **H** cell area measurements. Images were captured at 20 × magnification. Statistical significance was calculated using Student’s unpaired T-test (Fig. 6C and D), or one-way ANOVA with Dunnett's post hoc test relative to Dox + Ang II-treated animals (Fig. 6G and H). n = 3 animals in each group. ns, non-significant. Scale bar = 100 µm * p < 0.05, ** p < 0.01, **** p < 0.0001

To evaluate the protective effects of GDF15 against angiotensin II-induced cardiorenal stress, mice were surgically implanted with osmotic pumps to infuse angiotensin II along with intraperitoneal injection of saline (control) or GDF15 (Fig. 6A–D). In the angiotensin II-treated group, hypertensive remodeling was evidenced by cardiomyocyte hypertrophy quantified as increased cross-sectional area, irregular fibre alignment, and interstitial collagen deposition reflecting maladaptive extracellular matrix remodeling (Fig. 6A–D). In the GDF15 pretreatment group, cardiomyocytes retained an intact myocardial architecture, and were organized into parallel arrays with preserved sarcomeric striation and uniform cellular morphology (Fig. 6A). Angiotensin II-induced cardiac fibrosis (Fig. 6B and C) and hypertrophy (Fig. 6D) were both significantly attenuated by GDF15.

To compare and determine the incremental effects of anthracycline toxicity and cardiovascular stress via the renin-angiotensin system [46, 47], mice were treated with saline (control), doxorubicin (Dox), doxorubicin with angiotensin II (Dox + Ang II), or doxorubicin with angiotensin II and GDF15 (Dox + Ang II + GDF15) (Fig. 6E–H). In the Dox only group, cardiomyocytes exhibited pronounced pathologic alterations, including cytoplasmic vacuolisation, nuclear pyknosis, and focal myofibrillar loss, accompanied by regions of cardiomyocyte dropout, indicative of direct cytotoxic injury. In the Dox + Ang II group, histologic analysis of heart tissue revealed extensive pathological changes associated with pressure overload and cytotoxic injury, including cardiomyocyte hypertrophy characterized by increased cross-sectional area, irregular fibre orientation, and interstitial fibrosis (Fig. 6E–H). GDF15 pretreatment mitigated these pathological alterations associated with angiotensin II and doxorubicin (Dox + Ang II + GDF15), and enabled preservation of myocardial morphology and tissue architecture to an extent comparable with that of the control group (Fig. 6E–H).

**Fig. 7:**
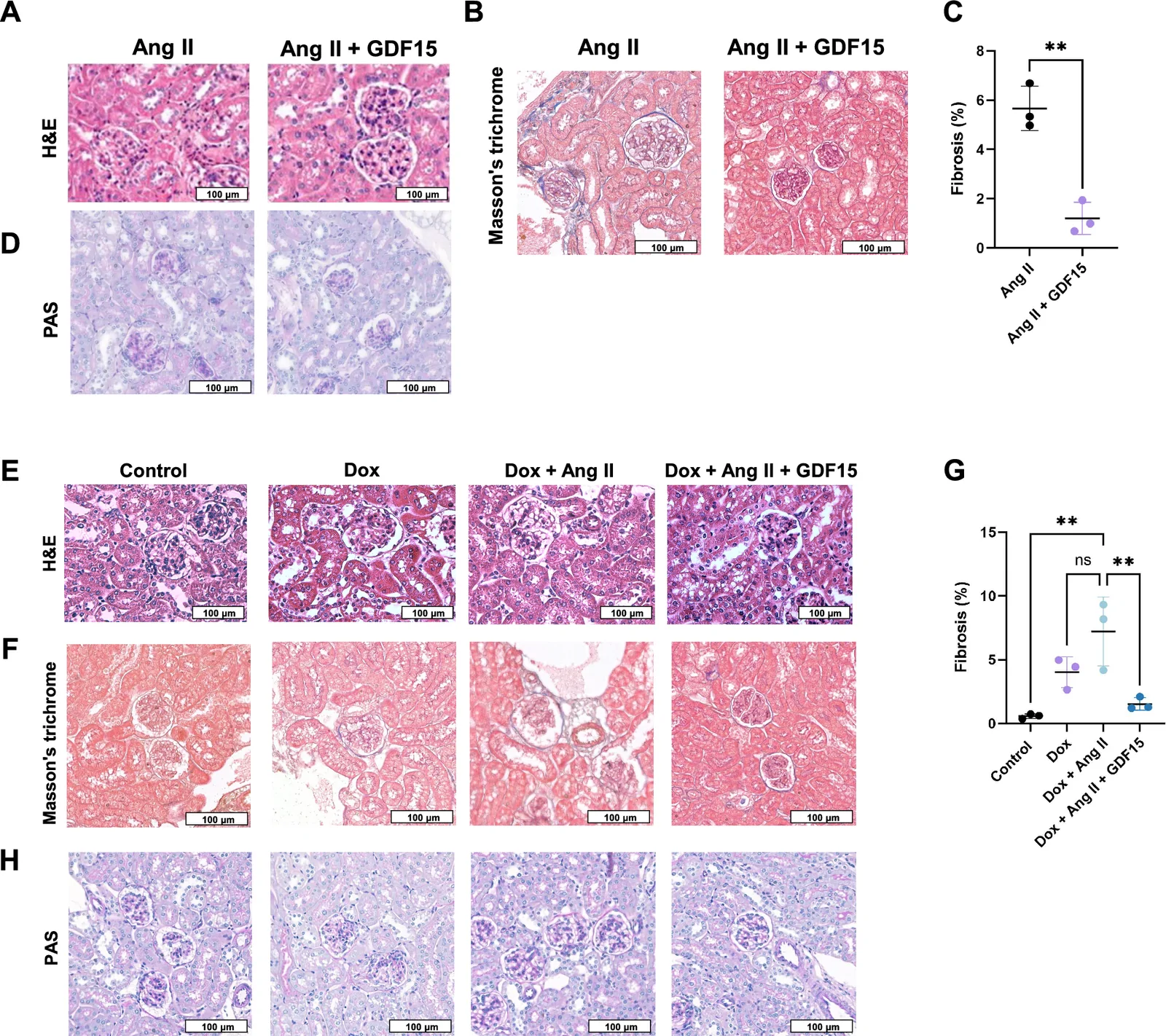
Renal damage induced by angiotensin II (Ang II) and doxorubicin (Dox) can be reduced by GDF15 pretreatment in mice. Mice were treated with Ang II ± GDF15. Control animals were injected with 0.9% saline. Kidney tissue sections were subjected to **A** H&E, and **B** and **C** Masson’s trichrome and **D** Periodic Acid-Schiff (PAS) staining. Mice were treated with vehicle control, Dox ± Ang II ± GDF15. Kidney sections were subjected to **E** H&E, **F** and** G** Masson’s trichrome staining and **H** PAS staining. Images were captured at 20 × magnification. Statistical significance was calculated using Student’s unpaired t-test (Fig. 7C), or one-way ANOVA with Dunnett’s post test relative to Dox + Ang II-treated animals (Fig. 7G). n = 3 animals were used for each experiment. ns, non-significant. Scale bar = 100 µm. ** p < 0.01

In the kidneys, Ang II alone promoted vascular wall thickening and fibrosis that were ameliorated by GDF15 (Fig. 7A–D). In animals treated with doxorubicin, the most extensive pathologic findings were seen in the Dox + Ang II group, with notable mesangial expansion, glomerular basement membrane thickening and renal tubular injury (Fig. 7E). These changes were markedly attenuated by GDF15. Analogous to results seen in the heart, GDF15 significantly reduced fibrosis and hypertrophy induced by doxorubicin and Ang II, as seen in the Dox + Ang II + GDF15 group (Fig. 7F–G). In summary, heart and kidney damage induced by doxorubicin and angiotensin II can be significantly reduced by GDF15 prophylaxis (Fig. 7).

## Discussion

This preclinical study is the first to characterize the protective effects of GDF15 pretreatment in comparison with dexrazoxane against doxorubicin in hESC-CMs in vitro and as a prophylaxis against heart and kidney injury and fibrosis in mice in vivo. We determined that 24-h pretreatment with recombinant GDF15 at 300 ng/ml optimally protected hESC-CMs in vitro. In our mouse model, prophylactic injection of GDF15 (0.5 mg per kg body weight) [26] 24 h prior to doxorubicin exposure was protective against cardiotoxicity. Similar to dexrazoxane, GDF15 significantly reduced doxorubicin-induced mitochondrial dysfunction and damage, cardiomyocyte apoptosis, heart and kidney injury, hypertension, and cardiorenal fibrosis. Collectively, our findings demonstrated the efficacy of GDF15 prophylaxis in protecting the heart and kidneys against doxorubicin with or without the escalated stress of angiotensin II.

Kempf et al. first demonstrated the protective effects of GDF15 in an ischemia–reperfusion injury animal model [48], several years after an observational study that reported an association between elevated plasma GDF15 levels and increased risks for cardiovascular events in human patients [49]. Zhang et al. later found that GDF15 was highly expressed in the conditioned cell culture media from induced pluripotent stem cell-derived mesenchymal stem cells that contributed to cardioprotection against anthracycline in mice [50]. However, no further study has shown direct evidence of recombinant GDF15 prophylaxis in protecting against anthracycline cardiotoxicity in vivo. We have advanced previous findings by demonstrating therapeutic feasibility in a mouse model the protective action of GDF15 against doxorubicin-induced cardiorenal toxicity and fibrosis.

Further insight can be gleaned from RNA sequencing revealing genes associated with the Wnt and SMAD signaling pathways [13] that mediate the protective effects of GDF15 against anthracycline. In particular, GDF15 has been shown antihypertrophic and antiapoptotic effects in CMs by activating Smad2/3 in mice [13]. Our findings extend the protective effects of GDF15 to countering doxorubicin toxicity via this pathway. There is scarce evidence in the literature linking GDF15 to Wnt signaling. However, Wnt signaling has been shown to regulate GDF15 expression in the context of steatohepatitis associated with metabolic dysfunction [51, 52]. The existence of a potential feedback loop, possibly involving cardiac metabolism and repair and the interaction between GDF15 and SMAD/Wnt signaling, awaits further investigation.

Available animal and human studies have focused on the pathological effects of anthracyclines on the heart, yet few have concomitantly investigated their detrimental effects on the heart and kidneys. The renin-angiotensin system has long been implicated in the exacerbation of anthracycline-induced cardiotoxicity, as pharmacological blockade of angiotensin receptor signaling has been associated with improved outcomes to varying extents in patients [6, 7]. Our demonstration of reduced cardiorenal damage and fibrosis by GDF15 prophylaxis represents an integrative approach to reducing the morbidity and complications associated with anthracycline.

The importance of the renin-angiotensin system and the central role of mitochondrial function and integrity in anthracycline-induced cardiorenal toxicity have been shown in experimental rodent studies [53, 54] but remain understudied [55]. Our findings demonstrated the capacity of GDF15 to counter doxorubicin and angiotensin II in the pathophysiology of anthracycline-induced cardiorenal toxicity in vivo. Beyond cardiorenal protection, GDF15 (also known as macrophage inhibitory cytokine 1 or MIC-1) has been shown to counterregulate inflammation and injury, and mediate immune tolerance [18, 56, 57]. These immune modulating effects may partly explain the attenuation of damage by anthracycline and angiotensin II. Tanaka et al. reported that doxorubicin-induced sterile inflammation was a key driver of reactive fibrosis preceding heart failure [58]. Findings from our RNA sequencing analysis supported this concept by showing GDF15 induction of immune regulatory genes that are known to ameliorate fibrosis including *CXCR7* [59, 60] and *TNFRSF11B* (also known as osteoprotegerin that inhibits interleukin-17-mediated myocardial fibrosis) [61]. Interestingly, *CXCL12* (also known as stromal cell derived factor 1 or SDF1), the gene encoding the ligand of CXCR4, is induced by GDF15 and may alter the balance of fibrogenesis while mediating the beneficial effects of CXCR4 and CXCR7 that are crucial for tissue repair [62], and may explain the complex action and cardiorenal benefits of GDF15 prophylaxis. The pathogenic roles of certain G-protein coupled receptors, including frizzled (FZD) and angiotensin II receptors, in cardiac remodeling are recognized [63] and validated by qPCR showing upregulated *FZD2* and *FZD4* expression (Fig. 4E). Further studies are needed to deepen mechanistic insights into the pathophysiology of renin-angiotensin activation in doxorubicin-induced toxicity and fibrosis.

There are several limitations to consider in this study. First, while hESC-CMs can serve as a model for studying human cardiac cells in vitro, they may not fully recapitulate the behavior of primary adult human cardiomyocytes [22, 64–66]. Culturing hESC-CMs for an extended period (60–120 days) as was done in this study, or using maturation markers to evaluate and characterize cellular phenotype in 3D culture [64, 67], may derive a more adult-like phenotype. Leveraging hESC-CMs has allowed us to better understand the optimal GDF15 pretreatment parameters against doxorubicin before experimentation in rodent models in vivo. Second, as clinical use of dexrazoxane has been associated with secondary hematologic malignancies [8], we have evaluated in MV4-11 cells the dose range in which GDF15 as pretreatment exerted optimal cardioprotective action against doxorubicin, and found no evidence of enhanced leukemia cell viability or protection from doxorubicin (Fig. 2). Instead, GDF15 alone at 300 or 1000 ng/ml significantly reduced MV4-11 cell viability but not numbers (cell count). Further studies are needed to characterize the effects of GDF15 on the biology and survival of different cancer types in the presence or absence of anthracyclines. Third, this study used young (10- to 12-week-old) male C57BL/6 mice for evaluating the prophylactic GDF15 regimen against doxorubicin with or without angiotensin II infusion. Generalizability of our findings can be further tested in and extended to female and aged mice, animals with different genetic background (e.g. BPH/2 J mice), and other models of induced hypertension (e.g. one-kidney one-clip model). Finally, as the mouse model used in this study was free of cancer, further investigations are needed to establish the protective efficacy and safety of GDF15 in the presence of active malignancy. The use of cancer-prone mouse strains, and animals implanted with different cancer types in conjunction with GDF15 prophylaxis may yield additional insight.

In conclusion, recombinant GDF15 pretreatment of hESC-CMs confers cytoprotection, and prophylactic administration of high-dose, pulsed GDF15 at 24 h prior to doxorubicin exposure can significantly reduce cardiorenal toxicity and fibrosis to a similar extent as dexrazoxane in a preclinical mouse model. The cardioprotective effects of GDF15 prophylaxis against doxorubicin toxicity appear to extend to heightened circulatory stress mediated by angiotensin II infusion.

## Supplementary Information

supplementary material file 1.

## Data Availability

The datasets generated during and/or analyzed during the current study are available from the corresponding author upon reasonable request.
