# Supplementary material for: Recombinant GDF15 prophylaxis attenuates cardiorenal toxicity and fibrosis induced by doxorubicin and angiotensin II
Source: Cell Biosci. 2026 Aug 16;16:121. doi: 10.1186/s13578-026-01633-9 (PMC13757164; doi:10.1186/s13578-026-01633-9)
Supplement: Supplementary file 1 [file 13578_2026_1633_MOESM1_ESM.docx]

**Supplementary Information**

Manuscript title: Recombinant GDF15 Prophylaxis Attenuates Cardiorenal Toxicity and Fibrosis Induced by Doxorubicin and Angiotensin II

Authors: Erik Fung, MD, PhD,^1,2,3,4,^* Hangqi Luo, PhD,^2,3,5,^*, Jack C.H. Chen, MSc,^6^ Wenjuan Zhu, PhD,^3,7^ Lihua Huang, PhD,^3^ Yidan Hao,^2,3^ Xiao-Ru Huang, PhD,^8^ Hui-Yao Lan, MD, PhD,^8^ Ellen N. Poon, PhD.^6,9,10^

Authors' affiliations and details: ^1^School of Medicine, Hong Kong University of Science and Technology, Hong Kong SAR; ^2^Laboratory for Heart Failure + Circulation Research, Li Ka Shing Institute of Health Sciences, and Gerald Choa Cardiac Research Centre, Faculty of Medicine, The Chinese University of Hong Kong, Hong Kong SAR; ^3^Centre for Cardiovascular Genomics & Medicine and Hong Kong Hub of Paediatric Excellence, The Chinese University of Hong Kong, Hong Kong Children’s Hospital, Hong Kong SAR; ^4^School of Public Health, Faculty of Medicine, Imperial College London, London, UK; ^5^Yale Cardiovascular Research Center, Section of Cardiovascular Medicine, Yale School of Medicine, New Haven, Connecticut, USA; ^6^School of Biomedical Sciences, Faculty of Medicine, The Chinese University of Hong Kong, Hong Kong SAR; ^7^Stanford Cardiovascular Institute, Stanford University, Stanford, California, USA; ^8^Inflammatory Disease Laboratory, Li Ka Shing Institute of Health Sciences, Faculty of Medicine, The Chinese University of Hong Kong, Prince of Wales Hospital, Hong Kong SAR; ^9^Hong Kong Hub of Paediatric Excellence (HK HOPE), The Chinese University of Hong Kong, Hong Kong SAR; ^10^Ministry of Education Key Laboratory for Regenerative Medicine, The Chinese University of Hong Kong, Hong Kong SAR. *These authors contributed equally.

Corresponding authors: Erik Fung ([eirkfung@ust.hk](mailto:eirkfung@ust.hk)), Ellen N. Poon ([ellen.poon@cuhk.edu.hk](mailto:ellen.poon@cuhk.edu.hk))

**Supplementary Table S1**

| Gene | Sequence (5’ to 3’) | T_m_  (^o^C) | Amplicon size (bp) |
| --- | --- | --- | --- |
| *NDUFAB1* | F: CGCGCAGGTTCCTGGTAG | 58.8 | 82 |
|  | R: GTCCTGGATGCCCTCTAACG | 58.6 |  |
| *SDHA* | F: TTGCAGCACAGGGAGGAATC | 57.5 | 129 |
|  | R: CCGTCATGTAGTGGATGGCA | 58.6 |  |
| *COX6B1* | F: CGGGGTGCCTTTAGGATTCA | 57.5 | 150 |
|  | R: TTCTGACAGCGGTGGAAGTC | 57.1 |  |
| *ATP5F1B* | F: CTGGTGTTGGTGAGAGGACC | 58.6 | 258 |
|  | R: CAGACACCTCTGAACCAGCC | 58.6 |  |
| *FZD2* | F: GTGCCATCCTATCTCAGCTACA | 58.6 | 195 |
|  | R: CTGCATGTCTACCAAGTACGTG | 58.9 |  |
| *FZD4* | F: CCTCGGCTACAACGTGACC | 58.1 | 155 |
|  | R: TGCACATTGGCACATAAACAGA | 54.3 |  |
| *WNT4* | F: AGGAGGAGACGTGCGAGAAA | 58.6 | 83 |
|  | R: CGAGTCCATGACTTCCAGGT | 58.6 |  |
| *GDF6* | F: CACGAGTACATGCTGTCAATCT | 56.4 | 96 |
|  | R: CGTATTAGCCGACTTGGAAGAC | 57.5 |  |
| *CXCL12* | F: ATTCTCAACACTCCAAACTGTGC | 56.4 | 88 |
|  | R: ACTTTAGCTTCGGGTCAATG | 53.5 |  |
| *CXCR7* | F: TCTGCATCTCTTCGACTACTCA | 56.4 | 130 |
|  | R: GTAGAGCAGGACGCTTTTGTT | 55.4 |  |
| *TNFRSF11B* | F: GGTCTCCTGCTAACTCAGAAAGG | 57.1 | 124 |
|  | R: CAGCAAACCTGAAGAATGCCTCC | 57.1 |  |

bp, base pairs; F, forward; R, reverse; T_m_, melting temperature
